# Trend Analysis of Homicide Mortality and Years of Life Lost in the South of Iran, 2004-2019

**DOI:** 10.34172/jrhs.2023.108

**Published:** 2023-03-30

**Authors:** Habibollah Azarbakhsh, Jafar Hassanzadeh, Seyed Sina Dehghani, Maryam Janfada, Mohammad Hossein Sharifi, Alireza Mirahmadizadeh

**Affiliations:** ^1^Student Research Committee, Shiraz University of Medical Sciences, Shiraz, Iran; ^2^Research Center for Health Sciences, Institute of Health, Department of Epidemiology, Shiraz University of Medical Sciences, Shiraz, Iran; ^3^School of Medicine, Shiraz University of Medical Sciences, Shiraz, Iran; ^4^Medical Records, Health Vice-chancellor, Shiraz University of Medical Sciences, Shiraz, Iran; ^5^Non-Communicable Diseases Research Center, Shiraz University of Medical Sciences, Shiraz, Iran

**Keywords:** Homicide, Mortality rate, Years of life lost, Joinpoint regression, Iran

## Abstract

**Background:** This study was conducted to determine the mortality rate and years of life lost (YLL) due to homicide in Fars province.

**Study Design:** This was a cross-sectional study.

**Methods: ** All data related to deaths due to homicide in Fars province were obtained from the Populationbased Electronic Death Registration System. Crude and age-standardized mortality rate, YLL, and YLL rate data were calculated and joinpoint regression was used to examine the trend.

**Results:** During 2004-2019, 2148 deaths due to homicide occurred in Fars province, and (1782 cases (83.0%) were men. The crude mortality rate in men decreased by 44.0% from 2004 to 2019, but a stable trend was observed in women. The total YLL due to homicide during these 16 years was 43230 (1.37 per 1000 people) in men and 8931 (0.29 per 1000 people) in women. According to the joinpoint regression analysis, the 16-year trend of the YLL rate due to premature mortality in men was decreasing, and the annual percent change (APC) was -4.00% (95% confidence interval [CI]: -6.60 to -1.20, *P*=0.008). However, women demonstrated stable trends in this respect, and APC was -0.50% (95% CI: -3.10 to 2.20, *P*=0.704).

**Conclusion:** The crude and standardized mortality rates and the number of YLL due to homicide in the study period had a significant decreasing trend in men but a stable trend in women. To control this issue, officials and policymakers should identify the areas of homicide and control its risk factors such as economic and social issues, drug addiction, and the state of violence.

## Background

 Homicide is defined an “unlawful death inflicted upon a person with the intent to cause death or serious injury”.^1^ It is one of the leading causes of death worldwide. Since the beginning of the 21st century, more people have died from homicide than war every year.^2^ It is predicted that by 2030, homicide will cause more deaths than infectious diseases such as tuberculosis.^3^ More than 464 000 people are annually killed worldwide. Homicide as a violent crime occurs in all countries regardless of political, religious, or economic background.^4^ Several factors play a role in increasing the incidence of homicide, including socio-economic pressures, cultural factors, and literacy.^5^ In 1996, the World Health Assembly (1996) called for violence research to be promoted as a public health research priority.^6^ In 2015, it was estimated that 51 000 adolescents globally died from homicide, which was far more than the number of adolescent deaths from armed conflict (30 000), including war and internal unrest ^7^.

 The fifth leading cause of death is 10-14 years old.^8^ The average homicide rate in the world is currently about 8 per 100 000 people per year. In the first decade of the 21st century, nearly 5 million people died due to interpersonal violence, making homicide a more important source of violent death than war.^9^ According to statistics, one person is killed almost every minute.^10^ Half of all homicides occur in countries with only 10% of the world’s population, while 3 billion people live in countries with low homicide rates.^11^ Variations in the global distribution of homicide rates — a reliable measure of levels of violence — are significant, ranging from a low in Japan (0.5 per 100 000) to over 50 per 100 000 in Côte d’Ivoire and El Salvador.^12^ In Iran, all previous studies on homicide were cross-sectional and were performed over one year, and the trend of changes in the rate of homicide in the country has not been investigated so far.^13^ The years of life lost (YLL) are an essential criterion for ranking the health status of society and observing their challenges. According to the report of the World Health Organization (WHO), the value of one year of life is three times more than the gross domestic product per capita of any country.^14^ Considering that no study has so far focused on determining the YLL due to homicide in Fars province, this study sought to measure the mortality rate and YLL due to homicide in Fars province.

## Methods

 This cross-sectional study was conducted in Fars province from 2004 to 2019. The data relating to all homicide deaths were extracted from the population-based Electronic Death Registration System by age, gender, and year of death based on the International Classification of Diseases (10th revision). In the mentioned system, all available sources have been used to detect, record, and collect death-related information. Repeated deaths were excluded from the study based on the father’s name and national number similarity. In addition, death cases suspected of homicide were excluded from the research, and only instances of definite homicide were included in the study.

###  Statistical analysis

 First, crude and age-standardized mortality rates (ASR) of homicide were calculated during the study years according to gender and the year of death. To calculate the crude mortality rate, the average population of Fars province was employed for each year. The standard population in 2013 for low- and intermediate-income countries was applied for standardization.

 Then, to calculate YLL, the standard life table was used, and life expectancy was determined for different age and gender groups, as well as the number of deaths due to homicide in each age and gender group based on the following relationship^15^:

 YLL_= _N Ce^(ra)^/ (β + r)^2^ [e ^-(β + r)(L + a)^[-(β + r) (L + a)-1] – e^–(β + r)a^]^16^

 Where N represents the number of deaths of a particular gender and age. In addition, N is the standardized life expectancy of the deceased at the same age and gender. Further, R and β are the discounting rate (0.03) and the contractual rate in calculating the age value (0.04), respectively. Furthermore, C is an adjusted fixed value (0.1658). Finally, a denotes the age at which death occurred and e is constant (2.71).

 First, the YLL was calculated according to 18 age groups, including 0-4, 5-9, 10-14, and the like, up to 85 years old, and then based on age groups, 0-4, 5-14, 15-29, 30-44, 45-59, 60-69, 70-79, and over 80 years are shown in a figure.

 The number of YLLs due to premature death due to homicide was analyzed using the YLL template of 2015, the WHO, in Excel spreadsheet software, version 2016.

 Joinpoint regression based on the log-linear model was used to examine the trend of crude and standardized mortality rates and YLL rates for different years. It describes changing trends over successive segments of time and the number of increases or decreases within each piece. The resulting line segment between joinpoints is defined by the annual percent change (APC) based on the line segment’s slope and the average annual percent change (AAPC). Joinpoint Regression Program 4.9.1.0 performed the analysis for the trend.

 The protocol of this study was reviewed and approved by the Ethics Committee of Shiraz University of Medical Sciences (Code: IR.SUMS.REC.1399.772). All study aspects were conducted according to the university’s code of ethics.

## Results

###  General homicide mortality rate and trend

 Overall, 2148 deaths due to homicide occurred in Fars province during 2004-2019, including 1782 cases (83.00%) in men, and 46.90% (1007 cases) were in the age group of 15-29 years.

Table 1 presents the crude mortality rate in men decreased by 44.00% from 6.39 (per 100 000 population) in 2004 to 3.61 per 100 000 population in 2019 (*P* = 0.007 for trend, AAPC = -3.80%). In women, the corresponding rate decreased from 1.18 (per 100 000 population) in 2004 to 1.02 (per 100 000 population) in 2019 (0.07%, *P* = 0.779 for trend, AAPC = 0.30%). Moreover, the standardized mortality rate in men decreased from 5.78 per 100 000 in 2004 to 3.18 per 100 000 in 2019 (*P* = 0.004 for trend, AAPC = -3.70%), and in women, it decreased from 1.12 per 100,000 population in 2004 to 0.92 per 100 000 in 2019 (*P* = 0.986 for trend, AAPC = -0.1%, Table 1).

**Table 1:** Crude and standardized mortality rate (per 100 000 population) and YLL due to homicide by gender and year in Fars province during 2004-2019

**Year**	**No. of death**		**Crude mortality rate**		**ASR (95% CI)**		**YLL**			
							**No.**		**(Per 1000)**	
	**Men**	**Women**	**Men**	**Women**	**Men**	**Women**	**Men**	**Women**	**Men**	**Women**
2004	119	21	6.39	1.18	5.78 (4.63, 6.93)	1.12 (0.61, 1.62)	2885	548	1.55	0.30
2005	111	15	5.99	0.84	5.11 (4.00, 6.23)	0.76 (0.33, 1.18)	2725	404	1.47	0.22
2006	80	16	4.32	0.88	3.94 (2.99, 4.89)	0.92 (0.49, 1.36)	1896	396	1.02	0.21
2007	158	20	8.45	1.9	7.38 (6.06, 8.69)	1.02 (0.54, 1.50)	3839	513	2.05	0.28
2008	147	22	7.79	1.18	6.68 (5.42, 7.94)	1.05 (0.56, 1.55)	3605	522	1.91	0.28
2009	133	30	6.98	1.59	5.89 (4.70, 7.08)	1.51 (0.94, 2.08)	3298	714	1.73	0.38
2010	161	21	8.37	1.10	6.75 (5.45, 8.04)	0.99 (0.52, 1.46)	4084	539	2.12	0.28
2011	107	27	5.50	1.40	4.44 (3.40, 5.49)	1.16 (0.64, 1.69)	2724	698	1.40	0.36
2012	89	27	4.52	1.38	3.98 (3.04, 4.91)	1.41 (0.89, 1.93)	1936	573	0.98	0.29
2013	114	27	5.71	1.37	4.66 (3.61, 5.71)	1.32 (0.80, 1.83)	2714	667	1.36	0.33
2014	123	34	6.08	1.71	5.33 (4.25, 6.40)	1.52 (0.94, 2.09)	2990	905	1.47	0.45
2015	115	18	5.61	0.89	4.80 (3.78, 5.83)	0.79 (0.38, 1.21)	2774	437	1.35	0.21
2016	83	20	3.99	0.98	3.51 (2.65, 4.37)	0.89 (0.45,1.32)	2022	504	0.97	0.24
2017	82	26	3.94	1.28	3.39 (2.54, 4.24)	1.14 (0.65, 1.63)	1985	585	0.95	0.28
2018	84	21	4.01	1.03	3.62 (2.76, 4.48)	1.03 (0.59, 1.47)	1970	496	0.94	0.24
2019	76	21	3.61	1.02	3.18 (2.37, 3.99)	0.92 (0.48, 1.36)	1783	430	0.84	0.21
Total	1782	366	5.66	1.18	4.83 (4.56, 5.09)	1.11 (0.98, 1.23)	43230	8931	1.37	0.29
*P *value	-	-	0.007	0.779	0.004	0.986	-	-	0.005	0.017

*Note*. YLL: Years of life lost; CI: Confidence interval; ASR: Age-standardized rate.

**Figure 1:**
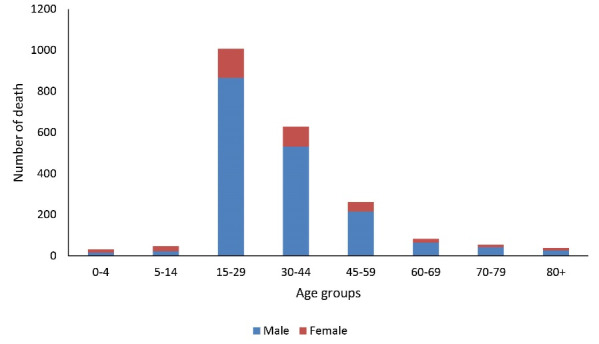
Number of homicide deaths by gender and age groups

 The highest and lowest deaths in both genders were in the age groups of 15-29 years and less than 5 years, respectively (Figure 1).

###  Temporal trends of homicide mortality by age groups

 In the 0-44 age group, the homicide mortality rate had a decreasing trend in men (AAPC = -3.50%, *P* = 0.043), while a stable trend was observed in women (AAPC = -1.90%, *P* = 0.387).

 However, there were decreasing trends in men (AAPC = -5.10%, *P* = 0.002) in the 45-59 age group, but stable trends in women (AAPC = 3.40%, *P* = 0.729).

 Based on the results, stable trends were observed in men (AAPC = -3.30%, *P*= 0.151) and women (AAPC = 8.10%, *P* = 0.301) in the age group of 60-74.

 Finally, there was a stable trend in men (AAPC = -0.50%, *P* = 0.971) and women (AAPC = 19.90%, *P* = 0.289) in the + 75 age group.

###  Temporal trends of homicide YLL rate

 The total YLL due to homicide during the 16-year study period was 43 230 (1.37 per 1000 people) in men and 8931 (0.29 per 1000 people) in women, and overall, 52 161 (0.83 per 1000 people) in both genders (men/women gender ratio, 4.84, Table 1). The average number of YLLs due to homicide was 24.3 and 24.4 years for men and women.

**Figure 2:**
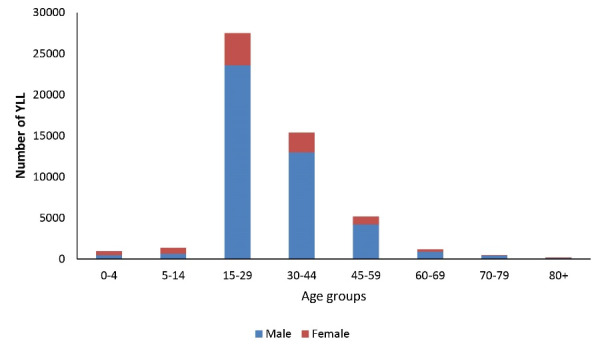
YLL due to homicide by gender and age groups. *Note*. YLL: Years of life lost

 The highest and lowest YLL in both genders were observed in the age groups of 15-29 years and under 5 years, respectively (Figure 2).

**Figure 3:**
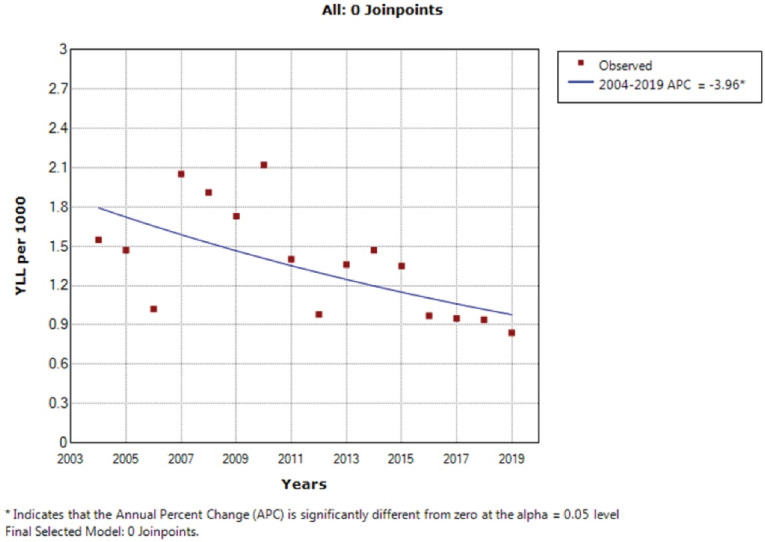
Trends in the YLL rate due to homicide in men during 2004-2019. *Note*. YLL: Years of life lost

**Figure 4:**
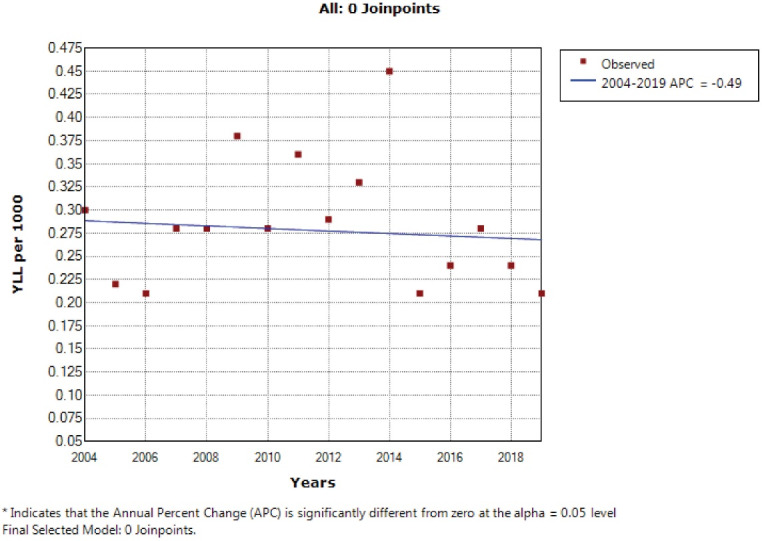
The trend of the YL rate due to homicide in women during 2004-2019. *Note*. YLL: Years of life lost

 According to the joinpoint regression analysis, the 16-year trend of the YLL rate due to premature mortality in men was decreasing. The APC was -4.00% (95% CI: -6.60 to -1.20; *P* = 0.008) and there were stable trends in women. According to the join point regression analysis, the 16-year trend of YLL rate due to premature mortality in men’s was decreasing. The APC was -4.00% (95% CI -6.60 to -1.20, *P* = 0.008) and stable trends in women, APC was - 0.50% (95% CI -3.10 to 2.20, *P* = 0.704) and decreasing trend for both sexes (total), APC was -3.40% (95% CI -5.80 to-0.90, *P* = 0.012). The model did not demonstrate any joinpoint; hence, the AAPC was the same as APC (Figures 3 and 4).

## Discussion

 The current study was conducted with the aim of investigating the mortality rate and YLL due to homicide in Fars province during 2004-2019. According to the authors, this study is the first one to examine the mortality rate and YLL due to homicide in 16 years in Iran. The results of this study showed that the crude and standardized mortality rates due to homicide in men decreased significantly from 2004 to 2019, while it had a stable trend in the same period of time in women. Moreover, the number of total YLLs due to homicide was higher in men than in women. The highest number of YLLs in both genders was in the age group of 15-29. The trend of the years of YLL homicide represented a decreasing trend in men, whereas it was stable in women. In a study conducted by Peres et al, the trend of the mortality rate due to homicide in different cities of the country was heterogeneous. Although there was a decreasing trend in some cities, an increasing trend was observed in other cities.^17^ In another study, mortality rates due to homicide decreased in Colombia and Mexico between 1996 and 2016. This trend decreased from 67 per 100 000 in 1990 to 34.3 per 100 000 in 2016 in Colombia, and it decreased from 18.8 per 100 000 in 1990 to 16.4 per 100 000 in Mexico in 2016,^18^ which is consistent with the results of the current study. In our study, the mortality rate due to homicide decreased from 3.8 per 100 000 in 2004 to 2.3 per 100 000 in 2019 (a 40.00% decrease).

 The results of another study performed in Iran^19^ revealed that the mortality trend of suicide and violence has been decreasing over the past three decades, supporting the findings of our study, whereas a relatively constant slope was reported in the rate of homicide in Russia between 2001to 2009.^20^ Therefore, homicide has different trends in various countries. Although violence appears to be a humanized, universal phenomenon, there is a great variation in the amount of violence in every society at a certain time. In some societies, violent attacks account for up to 60% of all deaths, and in other countries, they account for less than 0.05% of all deaths.^16^ In the present study, the mortality rate due to homicide was 5.7, 1.2, and 3.5 (per 100 000) in men, women, and both genders, respectively. Based on the results of another study,^12^ the homicide rate was 2.04, 11.73, and 17.45 in countries with high, medium, and low human development indexes (HDI), respectively (per 100 000). Compared to the findings of the study, the mortality rate due to homicide in Fars province was lower than in countries with a medium and low HDI, but higher than in continents with a high HDI. According to the reports of various studies in Iran and other countries regarding studying homicide, various factors such as gender, age, psychiatric disorders, climatic and ecological conditions, socio-economic level, cultural factors, poverty, individual violence, and family breakdown are involved in this regard.^10,13,21^ Nazari et al^13^ reported that the highest number of deaths and mortality due to homicide was in the age group of 15-29, and the crude and standardized rate of death was higher in men than in women, which is in line with the results of the current study. In our study, the highest number of deaths due to homicide was in the age group of 15-29, and the mortality rate was higher in men than in women.

 The existence of laws related to the prevention of all types of violence, including child abuse laws, laws against child marriage or the legal age of marriage for women or men, youth violence laws against school-licensed weapons or membership in gangs or criminal groups, sexual violence laws against sexual assault, victim laws, and compensations offered to victims, can affect the reduction of homicide in Fars province.^13^

 In a study conducted by Zavala-Zegarra et al, a constant trend was observed in the annual mortality rate due to homicide in men from 2001 to 2007 (APC = -0.80%), but this trend had a significant increase from 2007 to 2010 (APC = 11.10%). However, there was a non-significant constant trend during the ten-year study period in women (APC = -1.40%).^22^ Conversely, the results of this study contradict those of our study on the trend of the annual rate of homicide in men, which is consistent with this trend in women. In the current study, the mortality rate due to homicide in men significantly decreased during the 16-year study period (APC = -3.80%), but this trend was stable in women (APC = 0.30%). A progression in different aspects of health care, including the development of emergency and trauma care systems, may also play a significant role in decreasing homicide-related death rates. Differences in the biological features of males and females are described as the reasons for this trend.^23^

 In another study conducted in Iceland,^24^ the rate of YLL due to homicide was 50.4 per 100 000, which is lower than the results of our study (83 per 100 000). Veisani et al evaluated the YLL due to homicide during 2014-2018 and found that YLL due to homicide was more considerable in men and the age group of 15-29,^25^ which conforms to the results of our study. Homicides as a proportion of violent death remain high among younger age groups but abruptly decreased after the age of 45 years. Therefore, specific strategies to prevent gun violence are important to reduce the burden of violence. For example, when implemented well, street outreach strategies for high-risk youth in urban areas have shown promise for reducing gun violence.^26^

 The findings of this study demonstrated that the YLL measure can assess the burden of homicide and the effectiveness of efforts in the prevention of violent death. If resources are limited, YLL may especially help policymakers target premature death subpopulations and set top priorities for programs related to the prevention of violent death.^24^

 This study had some limitations. YLL was not evaluated throughout the whole of Iran due to the unavailability of the necessary data. The possibility of the incompleteness of the death registration system, as well as the misclassification of the death registration, could have affected the results of the study. The joinpoint regression is done as an ecological study. Moreover, multivariate analyses could not be yielded from the joinpoint regression program. On the other hand, this study was of high quality and had a strong study design, a large sample size, and the extensive time period of data analysis.

**Highlights**

- During 2004-2019, 2148 deaths occurred in Fars province due to homicide.
- The crude mortality rate in men decreased by 44.0% from 2004 to 2019, but there was a stable trend in this regard in females.
- The total YLL due to homicide was 43 230 (1.37 per 1000 people) in males and 8931 (0.29 per 1000 people) in females during the 16-year study period.

## Conclusion

 The crude and standardized mortality rates and the number of YLL due to homicide in the study period had a significant decreasing trend in men, but a stable trend in women. Although the decreasing trend in homicide in Fars province is good, it is not yet enough. To control this issue, officials and policymakers should identify the areas of homicide and control its risk factors such as economic and social issues, drug addiction, and the state of violence. Poverty, unemployment, social inequality, and incorrect tribal traditions are the other risk factors. To prevent youth violence, counseling, vocational training, family therapy, training health care workers in order to identify and refer youth at risk of violence, and policies pertaining to the reduction of the adverse effects of rapid social changes and dealing with gun violence among the youth are the other effective factors.

## Acknowledgments

 We would like to acknowledge the Health Vice-chancellor, Shiraz University of Medical Sciences.

## Competing Interests

 The authors declare that they have no competing interests.

## Funding

 None.
